# Maternal First-Trimester Vitamin B12 Levels as a Determinant of Infant Deficiency: A Single-Center Retrospective Study

**DOI:** 10.7759/cureus.95329

**Published:** 2025-10-24

**Authors:** Serap Ata, Ibrahim Tas

**Affiliations:** 1 Department of Pediatrics, University of Health Sciences, Ümraniye Training and Research Hospital, Istanbul, TUR; 2 Department of Pediatric Metabolism, University of Health Sciences, Ümraniye Training and Research Hospital, Istanbul, TUR

**Keywords:** breastfed infant, infancy, maternal vitamin b12, pregnancy, vitamin b12 deficiency

## Abstract

Background

Vitamin B12 deficiency in infancy may lead to growth retardation, anemia, and neurodevelopmental delay. Therefore, identifying maternal predictors is essential for early prevention. The significance of vitamin B12, particularly during pregnancy and early infancy, has been acknowledged. This study aims to identify the etiological factors in infants aged 9-12 months diagnosed with vitamin B12 deficiency.

Methodology

This study aimed to retrospectively evaluate the characteristics of infants aged 9-12 months with vitamin B12 levels ≤300 pg/mL and investigate the maternal vitamin B12 status during the first trimester.

Results

This retrospective study included 217 breastfed infants with vitamin B12 levels ≤300 pg/mL and a median age of 9.93 (9.0-12.0) months. Their median vitamin B12 level was 212 (100-300) pg/mL, with 43.8% (n = 95) having levels below 200 pg/mL, and 56.2% (n = 122) between 200 and 300 pg/mL. The median hemoglobin level was 11 g/dL, with anemia (<10.5 g/dL) present in 29% (n = 63) of infants; however, no cases of macrocytic anemia were observed. The first-trimester median vitamin B12 level in mothers was 246 (120-397) pg/mL, with 26% (n = 57) below 200 pg/mL, 50% (n = 109) between 200 and 300 pg/mL, and 23% (n = 51) above 300 pg/mL. Logistic regression analysis showed that infants of mothers with B12 <200 pg/mL had a 25.76 times higher risk of deficiency compared to the reference group (mothers with B12 >300 pg/mL) (relative risk = 25.760, 95% confidence interval = 8.616-77.013, p < 0.001). Among mothers, 62.6% with low B12 (<200 pg/mL) received treatment, predominantly with oral cyanocobalamin combinations, and the mean treatment duration was one month (n = 86, 94.5%).

Conclusions

Vitamin B12 deficiency is a common and underestimated public health problem in developing countries. This study demonstrates that maternal first-trimester vitamin B12 levels are the strongest determinant of deficiency in breastfed infants aged 9-12 months. These findings underscore the clinical importance of assessing maternal B12 status during pregnancy and even before conception as a primary preventive strategy.

## Introduction

Vitamin B12 (cobalamin) is essential for the development and function of the central nervous system and overall growth in infancy. Deficiency can lead to anemia, growth failure, and developmental delay, and, if left untreated, may cause irreversible neurological damage, particularly in infants and children of deficient mothers. Vitamin B12 (cobalamin) has important roles such as DNA synthesis, cell division, red blood cell formation, and myelination of the nervous system [1]. Due to rapid growth and development, these vitamins are especially important during infancy [2].

The concentration of vitamin B12 in breast milk depends on the mother’s condition and possibly on the mother’s intake of vitamin B12 during pregnancy and breastfeeding [3]. The content of vitamin B12 in breast milk decreases during the first four to six months of breastfeeding [4-6]. A decline in vitamin B12 levels has been observed in infants up to four to six months old [4,7]. Previous studies have reported that infants fed with formula or partially breastfed have a biomarker profile indicating a better vitamin B12 level compared to those exclusively breastfed. Additionally, studies in both high- and low-income settings show that breastfed infants in various age groups have lower cobalamin concentrations compared to non-breastfed infants, exhibiting higher levels of total homocysteine and methylmalonic acid (MMA), which are functional biomarkers of vitamin B12 status [8]. Typical symptoms of vitamin B12 deficiency include stunted growth, fatigue, hypotonia, refusal to eat, and delays in cognitive and motor development [9]. Population-based studies conducted in Nepal, India, Mexico, and Türkiye have reported low vitamin B12 levels in 30-40% of infants [10-13]. Assessing the prevalence of vitamin B12 deficiency during pregnancy is challenging due to physiological declines in serum levels and the limited reliability of biomarkers such as MMA and homocysteine. While some studies recommend newborn screening to detect moderate and severe vitamin B12 deficiency [14,15], others argue that current methods are not sufficiently reliable for large-scale implementation [16]. However, the German prospective study “Newborn Screening 2020” evaluated 26 additional metabolic disorders, including vitamin B12 deficiency, and found that maternal vitamin B12 deficiency was among the most frequently detected conditions, underscoring the importance of early screening in high-risk populations [15].

This study aimed to investigate the characteristics of infants aged 9-12 months diagnosed with vitamin B12 deficiency and the associated factors related to maternal vitamin B12 levels in the first trimester.

## Materials and methods

Study population and data collection

Between June 2023 and June 2024, healthy infants aged 9-12 months who presented for routine check-ups at the pediatric outpatient clinic of Ümraniye Education and Research Hospital, Istanbul, a tertiary care center, were retrospectively reviewed after being diagnosed with vitamin B12 deficiency. All necessary information regarding both the mother and the infant was collected from the hospital information system records.

Inclusion and exclusion criteria

Infants were eligible for inclusion if they met the following criteria: term birth, gestational age ≥37 weeks; serum vitamin B12 level ≤300 pg/mL; data completeness, availability of sufficient clinical and laboratory information in hospital records; recorded infant data, including age at presentation, birth weight, hemoglobin, hematocrit, mean corpuscular volume (MCV), ferritin, vitamin B12, folic acid, iron levels, and feeding type (breast milk and/or formula); and recorded maternal data, including documented first-trimester maternal vitamin B12 levels, treatment status, and information on folic acid and iron supplementation.

Exclusion criteria included preterm birth, missing or incomplete clinical or laboratory data, the absence of maternal first-trimester vitamin B12 levels, and lack of documentation regarding infant feeding type or hematological parameters.

Definitions of low vitamin B12 status and cut-off values

There are no age-specific cut-off values available for assessing vitamin B12 status in infants. When adult reference values are applied, serum vitamin B12 concentrations <148 pmol/L (<200 pg/mL) indicate probable deficiency, 148-258 pmol/L (200-350 pg/mL) indicate possible deficiency, and >258 pmol/L (>350 pg/mL) suggest that deficiency is unlikely [17]. According to the World Health Organization (WHO), vitamin B12 status in adults is classified as follows: >221 pmol/L (>300 pg/mL) is considered adequate, 148-221 pmol/L (200-300 pg/mL) is defined as low, and <148 pmol/L (<200 pg/mL) indicates deficiency. In the present study, both maternal and infant vitamin B12 levels ≤300 pg/mL were used as the cut-off value.

Serum vitamin B12 levels were measured from venous blood samples using chemiluminescent immunoassay or electrochemiluminescence immunoassay methods, which are standard automated techniques in clinical biochemistry laboratories.

For hemoglobin, anemia in children aged one month to five years was classified as mild (10-10.9 g/dL), moderate (7-9.9 g/dL), and severe (<7 g/dL), while the reference range for MCV was accepted as 70-84 fL, depending on age and gender.

To compare maternal and infant vitamin B12 status, both measurements were expressed in picograms per milliliter (pg/mL), allowing direct statistical comparison between groups. In our study, maternal first-trimester serum vitamin B12 levels were compared with infant levels obtained at 9-12 months to assess the correlation between maternal and infant B12 status.

Ethical approval

Ethics committee approval was obtained from the Istanbul Health Sciences University Ümraniye Training and Research Hospital Clinical Research and Ethics Committee (approval number: B.10.1.TKH.4.34.H.GP.0.01/300; date: October 03, 2024).

Statistical analysis

SPSS version 26 (IBM Corp., Armonk, NY, USA) was used for statistical analyses. Quantitative variables were described using mean, standard deviation, median, minimum, and maximum values, while qualitative variables were presented using descriptive statistical methods such as frequency and percentage. The Shapiro-Wilk test and box plots were used to assess the suitability of the data for normal distribution. The Mann-Whitney U test was applied to compare two groups with non-normally distributed variables, while the Kruskal-Wallis test was used for comparisons involving two or more groups. Spearman’s correlation analysis was used to assess relationships between variables, and the chi-square test was employed to compare qualitative data. Logistic regression analysis was performed to evaluate the effect of maternal vitamin B12 levels on low vitamin B12 levels in infants. All results were assessed within a 95% confidence interval, with significance set at p-values <0.05.

## Results

**Figure 1:**
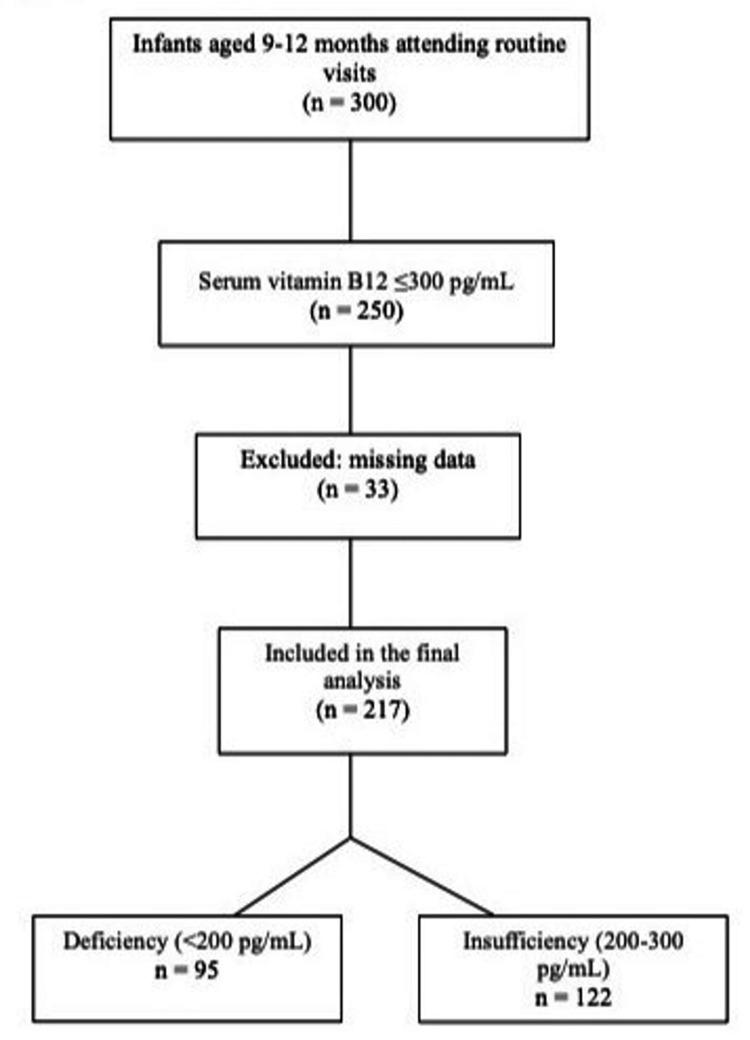
Flowchart illustrating patient selection.

A total of 217 infants with a median age of 9.9 months (9.0-12.0), a median birth weight of 3,155 g (2,340-4,380), and a vitamin B12 level of ≤300 pg/mL were included in the study (Figure 1). The mean ± SD vitamin B12 level in the infants was 207.1 ± 59.2 pg/mL, with 43.8% (n = 95) having levels <200 pg/mL and 56.2% (n = 122) having levels between 200-300 pg/mL. The mean ± SD hemoglobin value for infants was 11.1 ± 1.1 g/dL, with a threshold value for hemoglobin being 10.5 g/dL based on the month. Anemic hemoglobin levels (<10.5 g/dL) were present in 29% (n = 63) of the patients. Median ferritin levels were 28.77 ± 21.37 ng/mL, and only 22% had ferritin levels below the age-specific threshold of 12 ng/mL. The mean MCV levels were 74.57 ± 6.89 fL, and none of the patients had macrocytic anemia (Table 1). The mean folic acid level in infants was 13.16 ± 5.13 ng/mL, all of which were within the normal reference ranges.

**Table 1:** Demographic and laboratory characteristics of the infants and maternal vitamin B12 levels. Hb: hemoglobin; MCV: mean corpuscular volume; SD: standard deviation; n: sample size

	Value	n (%)
Age (months), median (minimum-maximum)	9.93 (9.0–12.0)	–
Birth weight (g), median (minimum-maximum)	3155 (2340–4580)	–
Plasma vitamin B12 (pg/mL), mean ± SD	207.1 ± 59.2	–
<200	–	95 (43.8)
≥200	–	122 (56.2)
Hemoglobin (g/dL), mean ± SD	11.1 ± 1.1	
<10.5	–	63 (29.0)
≥10.5	–	154 (71.0)
MCV (fL), median (minimum-maximum)	74.7 (57–78)	–
<70	–	50 (23.0)
70–84	–	167 (77.0)
Ferritin (ng/mL), median (minimum-maximum)	24 (3–121)	–
<12	–	48 (22.1)
≥12	–	169 (77.9)
Folic acid (ng/mL), median (minimum-maximum)	14 (9.8–20)	–
Nutrition status		
Breastfed	–	0
Breast milk + formula	–	0
Breast milk + supplementary food	–	217 (100)
Formula and/or supplementary food	–	0
Maternal vitamin B12 (pg/mL), median (minimum-maximum)	246 (120–397)	–
<200	–	57 (26.3)
200–300	–	109 (50.2)
>300	–	51 (23.5)

The median vitamin B12 level in mothers was 246 (120-397) pg/mL, with 26% (n = 57) having a level below 200 pg/mL, 50% (n = 109) between 200 and 300 pg/mL, and 23% (n = 51) above 300 pg/mL (Table 1).

All babies were exclusively breastfed and had not received any formula. At the time of the consultation, breastfeeding was ongoing, and all infants had begun complementary feeding (solid foods) alongside breastfeeding (Table 1). No significant difference was found between maternal B12 levels and infant birth weights (p = 0.294) (Table 2).

**Table 2:** Comparison of maternal vitamin B12 levels in terms of birth weight. Comparison using the Mann-Whitney U test.

		Birthweight (g)					
		Mean	SD	Median	Minimum	Maximum	P-value
Maternal B12 (pg/mL)	<200	3,158.07	351.50	3,150.00	2,500.00	4,300.00	0.294
	200-300	3,181.61	385.97	3,100.00	2,340.00	4,250.00	
	≥300	3,286.67	444.11	3,200.00	2,500.00	4,580.00	

Among babies of mothers with low maternal B12 levels (<200 pg/mL), 44.21% (n = 42) had B12 levels <200 pg/mL (Table 3). In contrast, among mothers with maternal B12 levels between 200 and 300 pg/mL, 50% of the infants (n = 61) had B12 levels >200 pg/mL. Only five of the mothers with B12 levels ≥300 pg/mL had babies with B12 levels <200 pg/mL (Table 4).

**Table 3:** Distribution of infant vitamin B12 levels according to maternal vitamin B12 status. Comparison was performed using the Mann-Whitney U test.

Maternal B12 (pg/mL)	Infant B12 <200 pg/mL (n, %)	Infant B12 200–300 pg/mL (n, %)	P-value
<200	42 (44.21%)	15 (12.30%)	<0.001*
200–300	48 (50.53%)	61 (50.00%)	
≥300	5 (5.26%)	46 (37.70%)	

**Table 4:** Comparison of maternal vitamin B12 and infant vitamin B12 levels. Comparison was performed using the Mann-Whitney U test.

Group	Mean ± SD (pg/mL)	Median (pg/mL)	Minimum (pg/mL)	Maximum (pg/mL)	P-value
Infant	205.37 ± 55.89	212.00	100.00	300.00	<0.001
Mother	243.49 ± 61.24	246.00	120.00	397.00	

The results of the logistic regression analysis, performed to analyze the effect of maternal B12 levels on low B12 levels in babies, are presented in Table 5. In the model, the effect of maternal B12 levels on the likelihood of infants having low B12 levels was assessed. In the group with mothers having B12 levels below 200 pg/mL, the risk of their babies having low B12 levels was 25.76 times higher compared to the reference group (B12 >300 pg/mL) (relative risk (RR) = 25.760, 95% CI = 8.616-77.013; p < 0.001). Babies born to mothers in the group with maternal B12 levels between 200 and 300 pg/mL were approximately 7.24 times more likely to have low B12 levels compared to the reference group (RR = 7.239, 95% CI = 2.670-19.627; p < 0.001) (Table 5). This finding suggests that even when the maternal B12 level is at a moderate level, it increases the risk of a deficiency in the baby’s B12 level. The constant term in the model (B = -2.219) was found to be significant (p < 0.001), indicating that in the reference group (mother’s B12 level >300 pg/mL), the likelihood of observing a low B12 level in babies was low (Exp(B) = 0.109).

**Table 5:** Assessment of risk factors affecting infant B12 deficiency through Logistic Regression Analysis The regression models; the effect of maternal B12 (pg /ml )levels on low B12 (pg /ml )levels in infants was analyzed using logistic regression

		B	S.E.	Wald	Sig.	Exp(B)	95% C.I.for EXP(B)	
							Lower	Upper
Step 1^a^	Mother B12 (>300)			34.865	< .001>			
	B12(<200)	3.249	.559	33.806	< .001>	25.760	8.616	77.013
	B12(200-300)	1.980	.509	15.131	< .001>	7.239	2.670	19.627
	Constant	-2.219	.471	22.210	< .001>	.109		

In 62.6% of the treated mothers (n = 57), B12 levels were below 200 pg/mL. Most of the treated mothers (70.3%, n = 64) were administered treatment with oral cyanocobalamin (1 mg cyanocobalamin, 250 mg vitamin B1, 250 mg vitamin B6 tablet), and the treatment duration was one month (n = 86, 94.5%) (Table 6).

**Table 6:** Maternal treatment status. po: per os; im: intramuscularly

Variable		n (91)	%
Maternal B12 (pg/mL)	<200	57	62.6
	200–300	31	34.1
	≥300	3	3.3
Type of treatment	Cyanocobalamin (po)	64	70.3
	Cyanocobalamin (im)	9	9.9
	Methylcobalamin (po)	18	19.8
Treatment duration	1 month	86	94.5
	2 month	5	5.5

## Discussion

This retrospective study was conducted in Türkiye, where vitamin B12 deficiency is commonly seen, to determine the etiological factors in infants aged 9-12 months whose vitamin B12 levels were tested routinely and found to be deficient. In the study, 43.8% of the babies had a vitamin B12 deficiency (<200 pg/mL) and 56.2% had insufficient vitamin B12 levels (200-300 pg/mL). All the babies were breastfed and had started complementary foods. The most important finding in the study is that 44.21% of mothers with B12 levels below 200 pg/mL during the first trimester were observed to have babies who still had B12 levels under 200 pg/mL at the ages of 9-12 months. Clinical symptoms of B12 deficiency in infants typically appear within the first six months of life, often accompanied by neurological findings such as irritability, apathy, and hypotonia [17,18].

This study’s patient group consisted of infants who were 100% breastfed. Previously, maternal B12 concentrations during pregnancy have been found to correlate at birth and in six-month-old infants [6,11]. The present research found that a low maternal B12 level in the first trimester correlates with the baby’s B12 level between the ninth and 12th months after birth. Previous studies have shown that if there is a deficiency of B12 in the mother, neonatal B12 stores are low, and when only breastfeeding, both the mother’s and baby’s B12 concentrations further decrease [7,11,19]. Vitamin B12 deficiency in infants, frequently observed due to the refusal of solid foods and difficulties in weaning children from milk, can facilitate prolonged exclusive breastfeeding [20]. Infants fed with formula are less likely to develop a B12 deficiency [21]. However, this result does not undermine the benefits of breast milk.

Low maternal serum B12 levels during the first trimester of pregnancy have been associated with low infant B12 levels and elevated MMA levels in newborn screening tests [15]. Therefore, it is important to identify B12 deficiency in women at the earliest stage, even before pregnancy [16]. In the maternal group of the present study, only 23.5% (n = 51) had B12 levels above 300 pg/mL, with the highest value being 394 pg/mL. Among the mothers receiving treatment, 62% had B12 levels below 200 pg/mL. In this research, only 34% of those with borderline deficiencies received treatment. Oral cyanocobalamin was preferred in 70% of cases, with 94% discontinuing treatment after approximately one month.

Folic acid supplementation is recommended for both pregnant women and those planning pregnancy to prevent neural tube defects. None of the infants in our study group had a folic acid deficiency. In our cohort, vitamin B12 deficiency during pregnancy was often treated with an orally administered tablet containing 1 mg cyanocobalamin, 250 mg vitamin B1, and 250 mg vitamin B6. Unfortunately, most pregnant women received only short-term treatment, and serum vitamin B12 control levels were not monitored. Additionally, measuring only serum B12 concentration during pregnancy may overlook clinically important cases [18].

It is believed that the concentration of vitamin B12 in breast milk depends on the mother’s status [3,4]. In two randomized controlled trials, maternal supplementation improved both breast milk concentrations and infant B12 status [22,23]. However, in these studies, supplementation began early in pregnancy and continued for 6-12 weeks postpartum, with high doses of vitamin B12 (50-250 µg/day). Unfortunately, there was insufficient information about maternal dietary intake in the present study; mothers reported using B12 supplements only for one to two months, and no control levels were available.

The WHO and US National Institutes of Health recommend higher daily cobalamin intake during pregnancy (2.6 µg vs. 2.4 µg/day) to support fetal neurological development [24,25]. Treatment of deficiency is similar to that in non-pregnant women and can be achieved with oral or parenteral replacement. When using 1,000 µg oral cobalamin daily, serum levels should be monitored to ensure adequate replacement [26].

Counseling on adequate vitamin B12 intake and supplementation during pregnancy, as well as considering B12 deficiency in the differential diagnosis of maternal anemia, are essential preventive measures [15]. Gramer et al. reported that in the German pilot project “Newborn Screening 2020,” maternal vitamin B12 deficiency was the most frequent finding among additional target disorders, with eight of 12 newly identified cases attributed to this condition, while the recall rate increased only marginally (0.1%) [27]. The inclusion of vitamin B12 deficiency in newborn screening has already shown benefits in Germany, where maternal deficiency was the most frequent condition detected in the extended panel.

Hematologic abnormalities are late symptoms of vitamin B12 deficiency [28]. Megaloblastic anemia caused by B12 and folate deficiency is rare in infancy. Laboratory examinations may reveal pancytopenia. Symptoms usually emerge between two and 12 months, but are rarely reported before three months [29]. None of the infants in the current study had megaloblastic anemia. All babies were given appropriate doses of iron prophylaxis, and all mothers had used folate replacement during pregnancy. Only 22% of the babies had low ferritin levels for their age, but all had normal folate values. This demonstrates the effectiveness of folate prophylaxis in the mothers and iron prophylaxis in the babies.

In Türkiye, it is recommended that all infants receive iron prophylaxis starting from the age of four to six months. The present study demonstrated no relationship between maternal B12 levels and infant birth weight. Inadequate maternal vitamin B12 status during pregnancy has been associated with adverse pregnancy outcomes such as spontaneous abortion, preterm birth, intrauterine growth restriction, low birth weight, and neural tube defects [29]. A systematic review and meta-analysis concluded that maternal vitamin B12 levels during pregnancy were not significantly related to birth weight [30]. In this study, maternal vitamin B12 status was not associated with other neonatal outcomes, including WHO z-scores, ponderal index, mid-upper arm circumference, or head circumference.

The limitations of this study include its retrospective design and the absence of additional biochemical markers such as total homocysteine, MMA, and serum holo-transcobalamin, which are frequently used to assess vitamin B12 status in both mothers and infants. Furthermore, neuromotor developmental assessments were not performed, and the study was confined to a narrow age range of 9-12 months, preventing evaluation of longitudinal changes in infant vitamin B12 levels. Another limitation is the lack of detailed data regarding maternal nutritional status, dietary habits, and pregnancy-related clinical conditions such as hyperemesis gravidarum, all of which could have influenced maternal vitamin B12 concentrations. As this was a retrospective study, such information was not consistently recorded in hospital data systems and therefore could not be analyzed. Additionally, this was a single-center study with a relatively small sample size, which restricts the ability to generalize the findings to national or regional levels. Broader multicenter studies including populations with diverse ethnic and dietary backgrounds are warranted to better characterize the epidemiology of vitamin B12 deficiency.

## Conclusions

In infants aged 9-12 months who are breastfed, maternal vitamin B12 levels during the first trimester represent the most critical determinant of infant vitamin B12 deficiency. The clinical significance of checking vitamin B12 lies in its potential to prevent neurodevelopmental delay, hypotonia, irritability, growth retardation, and megaloblastic anemia in infancy. If left untreated, vitamin B12 deficiency may lead to irreversible neurological impairment, cognitive delay, and behavioral dysfunction later in childhood. Therefore, early screening and supplementation during pregnancy and infancy play an essential preventive role. Although additional serum markers of vitamin B12 deficiency may not always be feasible due to economic limitations, they should be considered when possible, and the cut-off values for treatment should be reassessed. Given the high prevalence of vitamin B12 deficiency in Türkiye, establishing routine screening, preventive supplementation, and increased clinical awareness among pediatricians and obstetricians is strongly recommended. Moreover, integrating vitamin B12 measurement into newborn screening programs in countries where deficiency is common could provide substantial benefits with minimal additional burden.
